# Adaptation of Dominant Species to Drought in the Inner Mongolia Grassland – Species Level and Functional Type Level Analysis

**DOI:** 10.3389/fpls.2019.00231

**Published:** 2019-04-16

**Authors:** Yongzhi Yan, Qingfu Liu, Qing Zhang, Yong Ding, Yuanheng Li

**Affiliations:** ^1^Ministry of Education Key Laboratory of Ecology and Resource Use of the Mongolian Plateau, School of Ecology and Environment, Inner Mongolia University, Hohhot, China; ^2^Center for Biodiversity Dynamics in a Changing World, BIOCHANGE, Aarhus University, Aarhus, Denmark; ^3^Institute of Grassland Research, Chinese Academy of Agricultural Sciences, Hohhot, China

**Keywords:** drought, plant functional type, leaf economic spectrum, inner mongolia grassland, adaptation strategy

## Abstract

The adaptation of plants to drought through the adjustment of their leaf functional traits is a hot topic in plant ecology. However, while there is a good understanding of how individual species adapt to drought in this way, the way in which different functional types adapt to drought along a precipitation gradient remains poorly understood. In this study, we sampled 22 sites along a precipitation gradient in the Inner Mongolia grassland and measured eight leaf functional traits across 39 dominant species to determine the adaptive strategies of plant leaves to drought at the species and plant functional type levels. We found that leaf functional traits were mainly influenced by both aridity and phylogeny at the species level. There were four types of leaf adaptations to drought at the functional type level: adjusting the carbon-nitrogen ratio, the specific leaf area, the nitrogen content, and the specific leaf area and leaf nitrogen content simultaneously. These findings indicate that there is the trade-offs relationship between water and nitrogen acquisition as the level of drought increases, which is consistent with the worldwide leaf economics spectrum. In this study, we highlighted that the leaf economic spectrum can be adopted to reveal the adaptations of plants to drought in the Inner Mongolia grassland.

## Introduction

Arid and semi-arid regions occupy approximately 45% of the Earth’s land area and feed 38% of its population but also include some of the most vulnerable ecosystems and water resource systems (Narisma et al., 2007). In these regions, plant diversity maintains the ecosystem processes and functions and also affects ecosystem services (Balvanera et al., 2006). Water as the main limiting factor in these regions is one of the most important abiotic stresses influencing the survival, growth and distribution of plants. Therefore, the adaptation of plants to drought has always been a hot topic in ecological research (Chaves et al., 2002; Shavrukov et al., 2017). The characteristics of leaves are essential for the adaptation of plants to environmental change as leaves not only exhibit strong sensitivity and plasticity to spatial and temporal changes in the environment but can also improve the adaptability of plants through self-regulation (Picotte et al., 2009; Zhang et al., 2018). Therefore, the adaptation of plants to drought through the adjustment of leaf morphology has received much attention (Stropp et al., 2017).

Plant leaf exhibit both morphological and anatomical adaptations to drought. For example, plants that have grown under water-deficient conditions for a long period of time produce thickened, smaller leaves with a cracked appearance and a small specific leaf area, among other characteristics. Indeed, the leaves of some xerophytes become fleshy or even degenerate into rods. Such characteristics are conducive to reducing transpiration and promoting more effective heat dissipation (Bosabalidis and Kofidis, 2002; Stropp et al., 2017). In addition, plant leaves exhibit physiological and stoichiometric changes under drought conditions. For example, the photosynthetic rate may decrease as the level of drought increases (Tanaka and Shiraiwa, 2009). Furthermore, changes in the photosynthetic rate will further affect the uptake and recycling of nutrient elements and eventually lead to changes in the ecological stoichiometric characteristics of the leaves (Yuan et al., 2006; Zhang et al., 2018). He and Dijkstra (2014) experimentally showed that drought has a significant negative effect on plant nitrogen and phosphorus contents and a positive effect on the plant nitrogen-phosphorus ratio, while other experiments have shown that moderate drought stress may increase the uptake of nitrogen and decrease the growth rate of plants, resulting in a decrease in the carbon-nitrogen ratio (Lu et al., 2009).

Wright et al. (2004) proposed the concept of “leaf economics spectrum” (LES), which is a universal spectrum consisting of key leaf chemical, structural and physiological traits. At one end of the LES are species that have a “rapid investment-return” strategy, i.e., species with high leaf nitrogen contents, photosynthetic rates and respiration rates, short life spans and low specific leaf weights, while at the other end are species that have a “slow investment-return” strategy, i.e., species with long life span, large specific leaf weight, low nitrogen content, photosynthetic rate and respiration rate (Osnas et al., 2013). The resource trade-offs strategy is an important mechanism for LES, whereby plants that invest more resources in a particular functional trait will inevitably reduce the input of resources into other traits due to the total amount of resources that are available to the plants being limited, and such a trade-offs strategy has provided a mechanism by which plants can adapt to the environment in different geographical regions and ecosystems (Wright et al., 2005; Shipley et al., 2006; Ocheltree et al., 2016). Zhang et al. (2017) found that the trade-offs strategy of hydrophytes functional traits coincided with the worldwide LES. Abrahamson (2007) found that both *Serenoa repens* and *Sabal etonia* used a trade-offs strategy for functional traits under drought conditions, such as miniature plant morphology, a relative decrease in leaf size, number and photosynthetic yield, and prolonged leaf longevity, observing significant correlations among these traits and the formation of a continuously changing trade-offs strategy zone with the same plant species and homologous plants growing in shady or humid areas. Lohbeck et al. (2013) found that the trade-offs strategy is important for functional composition changes with succession in the dry and wet forest.

Studies that focus on characteristics of leaves of individual single species under different environmental conditions are important for revealing specific adaptive strategies at the species level (Zhu et al., 2012; Ramirez-Valiente et al., 2015; Liu et al., 2018). However, to generalized the plant adaptive strategies and the effects of global climate change on ecosystems, it is essential to group vegetation that is ecologically similar. The plant functional type is an assemblage of plants that share a set of key functional traits, respond to the environment in equal ways and play similar roles in the main ecosystem processes, (Chapin et al., 1996; Semenova and van der Maarel, 2000) and it has been shown that adaptive mechanisms often vary at the species and plant functional type levels (Domínguez et al., 2012). Although the environment is considered a key factor affecting leaf functional traits at the species level (Albert et al., 2010; Bu et al., 2017), the phylogenetic relationship among different species should also be considered because species with similar phylogenetic relationship may have similar functional traits (Kaplan and Pigliucci, 2001; Webb et al., 2002; Grether, 2005; Losos, 2008). In contrast, some studies have shown that plant functional types are the consequence of the adaptive processes of plants rather than branching processes in plant lineage (Pie and Weitz, 2005; Silva and Batalha, 2011). Thus, plant functional types can represent different adaptive strategies and may thus represent an efficient tool for revealing patterns of adaptation to environmental change (Ian and Wolgang, 2010). Moreover, understanding what adaptive strategies allow plants to successfully pass through the filter along an environmental gradient is of major importance in ecology. Moreover, understanding what adaptation strategies allow plant to pass through filter along the environment gradient successfully is a major issue in ecology.

The Inner Mongolia Plateau is a typical arid and semi-arid region with a large spatial precipitation gradient, making it an ideal place to study the adaptive mechanisms of plants to drought (Wu et al., 2015). In this study, we sampled 22 sites along the precipitation gradient of the Inner Mongolia grassland and measured eight leaf functional traits in 39 dominant species to address the following questions: (1) How are plants adapted to drought by modifying their leaf functional traits at the species and plant functional type levels? (2) Is the LES existing and applied to the adaptation of plants to drought in the Inner Mongolia grassland?

## Materials and Methods

### Study Sites

**FIGURE 1:**
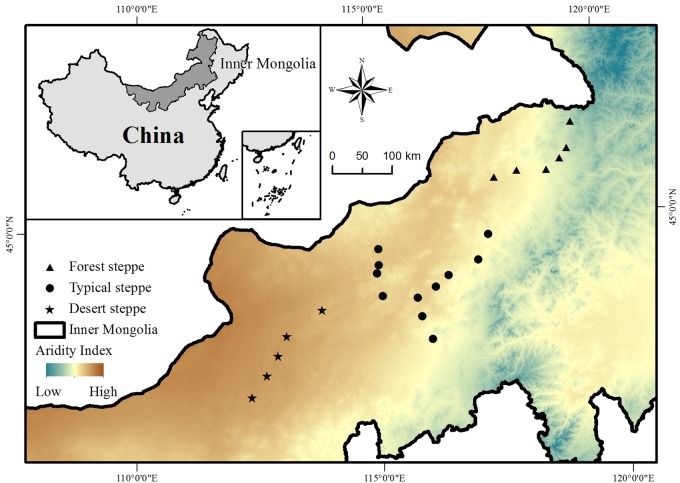
Study area and sites.

This study was conducted across the entire area of the Inner Mongolia grassland in northern China, which stretches from 41.31°N to 50.78°N and 108.16°E to 120.39°E and has an elevation ranging from 532 to 1725 m above sea level (Figure 1). The typical landforms in this region include gently rolling plains, tablelands, and hills. The mean annual temperature ranges from -3.0 to 6.7°C and the mean annual precipitation varies from approximately 150 to 450 mm, decreasing from the southeast to the northwest (Inner Mongolia-Ningxia Joint Inspection Group of Chinese Sciences of Academy, 1985). The aridity at each study site (see below) was calculated as [1 - precipitation/potential evapotranspiration] (Delgado-Baquerizo et al., 2013). Along the climate gradient from the relatively humid southeast to the relatively dry northwest, there is a succession of soil types from chernozems to chestnut and calcic brown soils, and a succession of habitat types from forest steppe through to typical steppe and desert steppe grassland (Figure 1).

### Data Collection

We surveyed 22 sites across the Inner Mongolia grassland in August 2017 during the peak period of aboveground biomass for the three main vegetation types: forest steppe, typical steppe, and desert steppe. The study sites had been banned grazing for over 3 years to minimize the potential effects of grazers and other disturbances. Five 1 × 1 m^2^ quadrats were randomly set at each site and species within each quadrat were recorded. The functional traits were then recorded for all healthy and pest-free plants of each of the species found at each site following the standard measurement methods for plant functional traits (Cornelissen et al., 2003). Five individuals of each species with good vigor and no pests or diseases were selected in each quadrat and eight functional traits that are sensitive to environmental change were measured on three intact leaves per plant. The leaf area (LA) was measured using a leaf area meter (LI-3100 Area Meter; LI-COR, Lincoln, United States). The leaves were then oven-dried at 60°C to obtain the leaf dry weight (LDM). The specific leaf area (SLA) was calculated based on the leaf area and leaf dry weight. The leaf nitrogen and carbon contents (LNC, LCC) were measured with an elemental analyzer (Euro Vector EA3000; Milan), and the carbon-nitrogen (LC:N) and nitrogen-phosphorus ratios (LN:P) calculated. Finally, the total leaf phosphorus content (LPC) was determined using the ammonium molybdate spectrophotometric method (Bowman, 1988). The average values of the functional traits of each species at their respective sites were used in this study.

### Statistical Analyses

We selected 39 species that had more than three occurrences across the entire sites for analysis. At the species level, we used the generalized linear model (Poisson distribution) to analyze the variation of eight plant functional traits that explained by climate (aridity) and phylogeny (genus). To analyze the respective and common interpretations of climate and phylogeny for each functional trait, we constructed three generalized linear models that included the trait as the response variable and aridity, genus, both aridity and genus as the explanatory variables, respectively. Thus, the *R*^2^ values of the first and second models represented the amount of variation in a particular functional trait that explained by aridity and phylogeny, respectively, while the difference in the *R*^2^ values between the sum of the first and second models and the third model indicated the combined effect of aridity and phylogeny on the each functional trait.

To analysis the adaptation of these species to drought at the plant functional type level, we first defined the plant functional types using cluster analysis (Semenova and van der Maarel, 2000). We constructed the clustering tree based on the Euclidean distance calculated using the eight functional traits of each species, each of which was first standard (Chapin et al., 1996; Montes-Pulido et al., 2017). Once the 39 species had been divided into functional groups, one-way ANOVA and multiple comparisons were performed to examine the differences in the eight functional traits between the functional types. To evaluate how each of the plant functional types adapted to drought, the direct relationship between aridity and the proportion of each functional type at a site was assessed by Pearson correlation analysis and ordinary least squares regression analysis, in which the proportion of plant functional types was represented by the ratio of the number of species which belongs to a given functional type to the total number of species at the site. Finally, to reveal how the different plant functional types successfully passed through the drought filter along the precipitation gradient and adapted to drought in the Inner Mongolia grassland, we used structural equation model to assess the causal relationships between aridity, the mean functional traits of the plant functional types and the proportion of plant functional types at a site using standardized path coefficients.

The generalized linear model, Pearson correlation analysis, one-way ANOVA, ordinary least squares regression and cluster analysis were conducted in R version 3.5.1. The structural equation model was conducted using the AMOS software. Shapiro–Wilk test was used to test the normality of the data before analysis and a log-normal transformation was used to normalize any variables that did not conform to the normal distribution.

## Results

### Effects of Aridity and Phylogeny on Variation in Plant Functional Traits at the Species Level

Both aridity and phylogeny had significant effects on plant leaf functional traits, with variation in the leaf area, specific leaf area and leaf carbon-nitrogen ratio being explained by both factors together, and variation in the leaf carbon content being explained only by aridity (Table 1).

**Table 1:** Amount of variation in the leaf functional traits explained by phylogeny and aridity at the species level.

Plant functional trait	Phylogeny (%)	Aridity (%)	Phylogeny and aridity (%)
Single leaf area	22.88^∗^	12.51^∗^	2.26
Dry weight of single leaf	24.76	7.20	2.05
Specific leaf area	0.40^∗^	22.32^∗^	0.39
Leaf carbon	0.23	1.99^∗^	0
Leaf nitrogen	1.70	5.00	0.98
Leaf phosphorus	0.14	0.99	0.11
Leaf carbon-nitrogen ratio	2.57^∗^	9.78^∗^	1.44
Leaf nitrogen-phosphorus ratio	0.04	1.55	0.04


*^∗^*p <* 0.05.*

### Classification of Plant Functional Types

**FIGURE 2:**
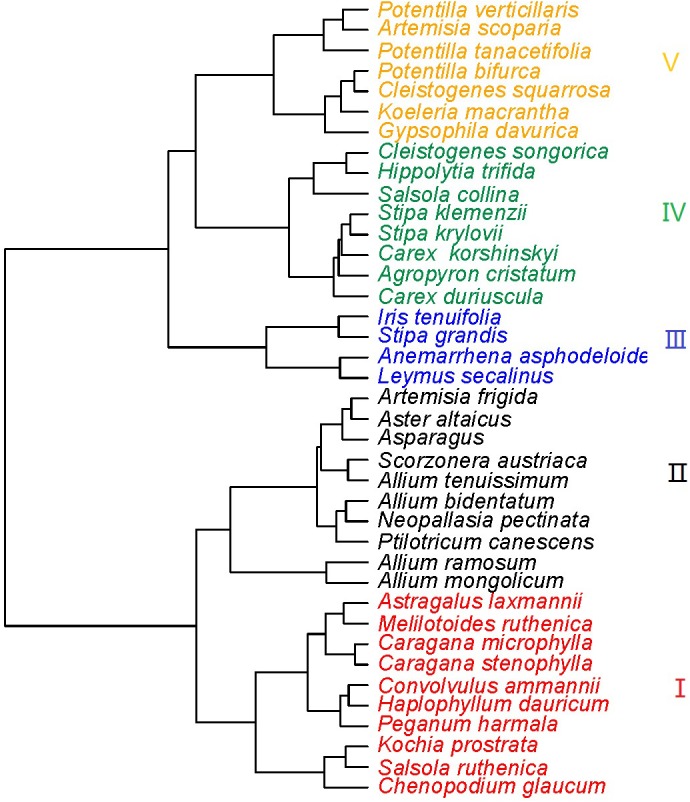
Cluster diagram of the eight species functional traits (Red, functional type I; black, functional type II; blue, functional type III; green, functional type IV; orange, functional type V).

**FIGURE 3:**
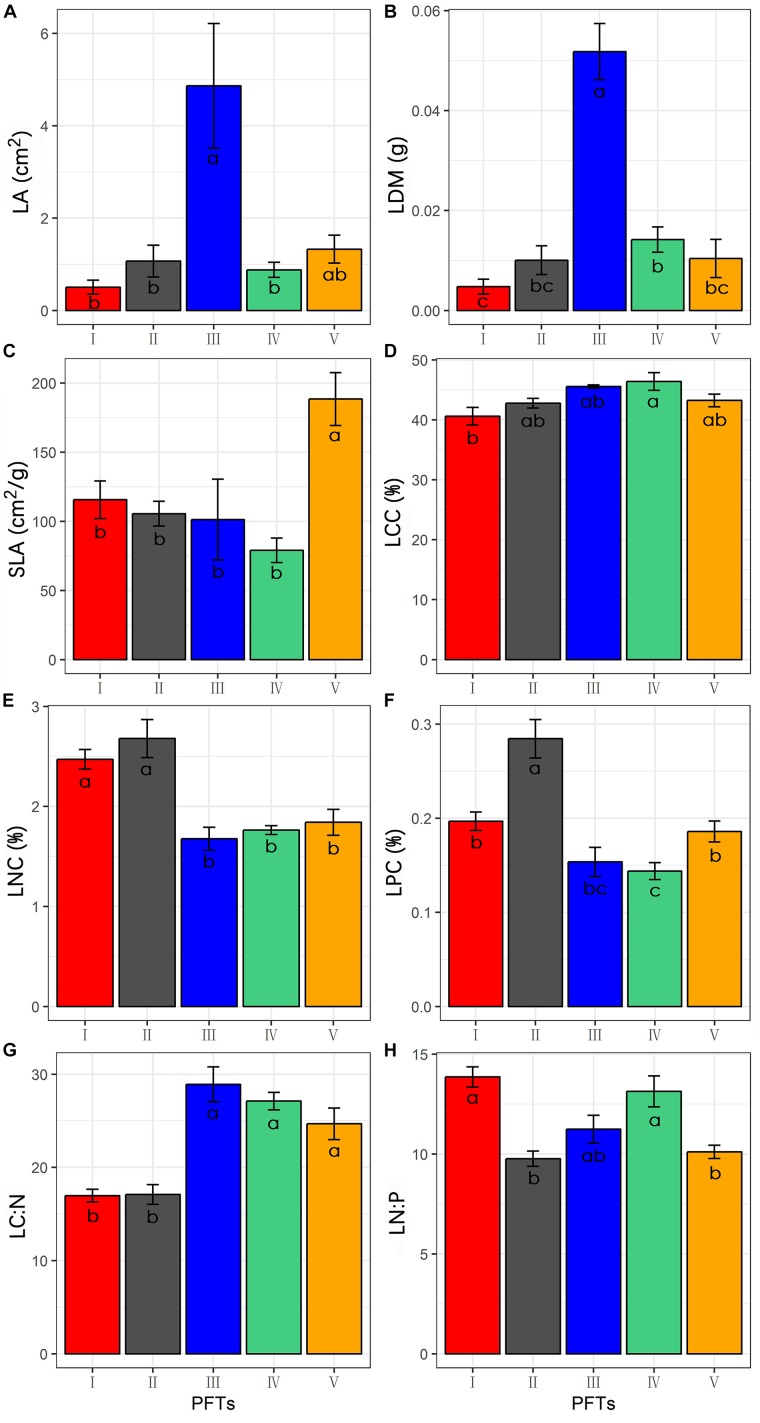
Differences of functional traits among the five functional types (Data are the means and SE of eight functional traits in each of the five functional types). **(A)** Difference of the leaf area; **(B)** difference of the leaf dry weight; **(C)** difference of the specific leaf area; **(D)** difference of the leaf carbon content; **(E)** difference of the leaf nitrogen content; **(F)** difference of the leaf phosphorus content; **(G)** difference of the leaf carbon-nitrogen ratio; **(H)** difference of the leaf nitrogen-phosphorus ratio.

The 39 plant species analyzed could be divided into five functional types based on the eight functional traits measured (Figure 2). The differences in the eight functional traits among the five functional types are shown in Figure 3. Functional type III had a significantly higher single leaf area (Figure 3A) and leaf dry weight (Figure 3B) than the other four types, while functional type V had a significantly higher specific leaf area (Figure 3C). In terms of the leaf carbon content, functional type IV had significantly higher levels than functional type I but there was no significant difference among the other three functional types (Figure 3D), while for leaf nitrogen content, functional types I and II had remarkably higher levels than the other three functional types (Figure 3E). For the leaf phosphorus content, functional type II had much higher levels than the other four functional types and functional type IV had the lowest level (Figure 3F). With respect to the leaf carbon-nitrogen ratio, functional types III, IV, and V had higher values than functional types I and II (Figure 3G), while for the leaf nitrogen-phosphorus ratio, functional types I and IV had much higher values than functional types II and V, with no significant difference being observed between functional type III and any other functional type (Figure 3H).

### Adaptation of Plants to Drought at the Functional Level

**FIGURE 4:**
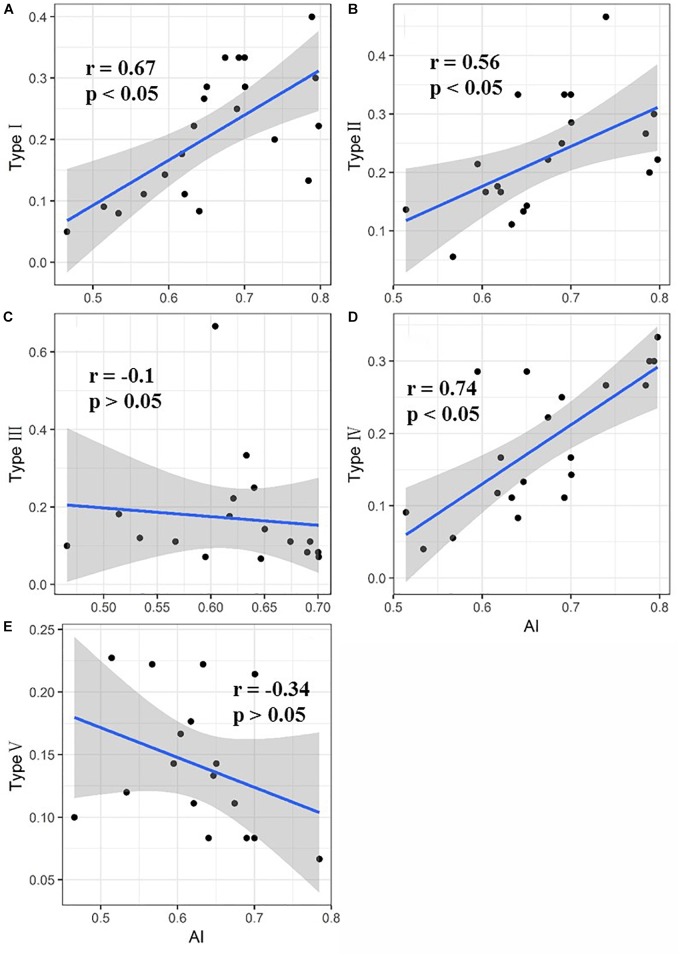
Direct response of each of the five plant functional types to drought (Values for types I, II, III, IV, and V represent the proportion of each functional type in the sample plots. The shaded areas indicate the 95% confidence intervals). **(A)** Direct response of plant functional type I to drought; **(B)** direct response of plant functional type II to drought; **(C)** direct response of plant functional type III to drought; **(D)** direct response of plant functional type IV to drought; **(E)** direct response of plant functional type V to drought.

**FIGURE 5:**
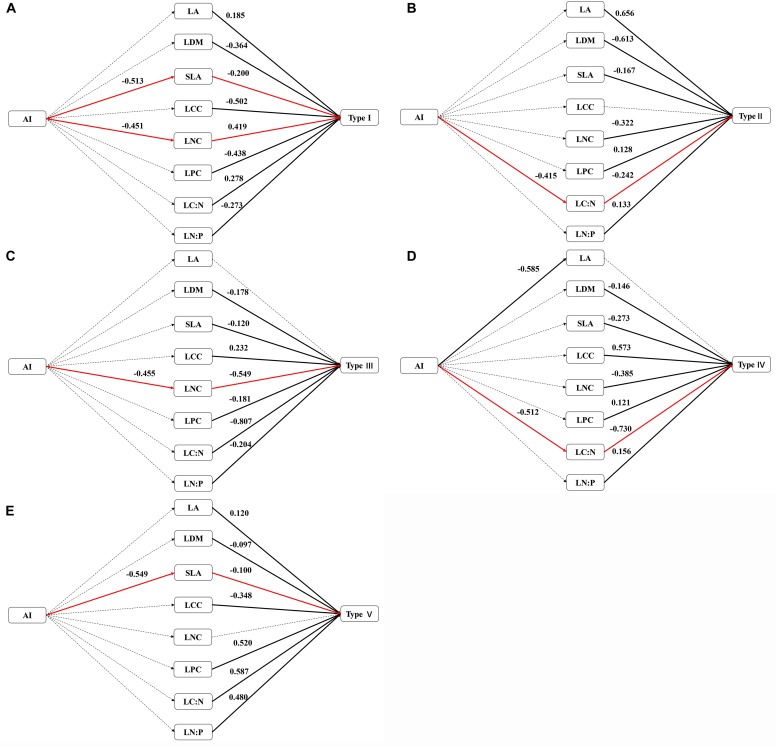
Response paths of different functional types to drought (Dotted lines represent non-significant paths (*p* > 0.05), black lines represent significant paths (*p* < 0.05) and red lines represent significant paths across AI-Functional traits: the proportion of functional types; values for types I, II, III, IV, and V represent the proportion of each functional type in each site). **(A)** Response path of type I to drought; **(B)** response path of type II to drought; **(C)** response path of type III to drought; **(D)** response path of type IV to drought; **(E)** response path of type IV to drought.

The relationship between the proportion of each functional type at each site and the aridity is shown in Figure 4. The proportion of functional types I, II, and IV in the sample plots significantly increased as the aridity increased, while the proportion of functional types III and V tended to decrease but not significantly. Functional type I plants exhibited a simultaneous reduction in leaf area and leaf nitrogen content with the increase of aridity (Figure 5A), while functional type II plants reduced the carbon-nitrogen ratio in their leaves (Figure 5B), functional type III plants increased their leaf nitrogen content (Figure 5C), functional type IV plants reduced the leaf carbon-nitrogen ratio in their leaves (Figure 5D) and functional type V plants reduced the leaf nitrogen content (Figure 5E) in response to drought.

## Discussion

In the present study, we demonstrated that aridity significantly affected the plant specific leaf area, leaf carbon content and leaf carbon-nitrogen ratio at the species level in the Inner Mongolia grassland, which is consistent with the findings of previous studies which found that plants can adapt to drought by regulating their leaf functional traits (Wright et al., 2004; Jung et al., 2014; Valencia et al., 2015). Some studies have also demonstrated that phylogeny would impact the functional traits, i.e., species with close phylogenetic relationships have similar functional traits (Ackerly, 2009; Burns and Strauss, 2012). Similarly, we found that the single leaf area, specific leaf area and leaf carbon-nitrogen ratio were significantly affected by phylogeny at the species level, suggesting that these three functional traits may be “conserved traits” that have a have strong phylogenetic signal (Blomberg et al., 2003). Our finding that these three functional traits were significantly affected by both phylogeny and drought suggests that species with adaptive functional traits are selected for and retained under long-term, strong environmental filtering, supporting the findings of previous studies (Grether, 2005; Liu et al., 2013). Further analysis showed that the variation in leaf area could be better explained by phylogeny than by climatic factors, while the specific leaf area and leaf carbon-nitrogen ratio were mainly affected by climatic factors, demonstrating that the importance of phylogeny and climatic factors differs between different functional traits.

Many studies have reported that plants adapt to drought conditions by adjusting leaf functional traits in various ways, such as the leaf dry matter content, specific leaf area, leaf carbon, nitrogen and phosphorus contents, and leaf stoichiometric ratio (Hameed et al., 2012; Stropp et al., 2017). In the present study, we found that plants in the Inner Mongolia grassland adapted to drought mainly by adjusting their leaf carbon-nitrogen ratios, specific leaf area and leaf nitrogen contents (Figure 5). Plant functional types II and IV showed the same adaptive strategy to drought, by decreasing the carbon-nitrogen ratio and then increasing in proportion in the sample plot (Figure 4, 5). The carbon-nitrogen ratio of plant leaves reflects the growth rate of plants to a certain extent, as a large amount of proteins, chlorophyll and rRNA are required for plant growth which, in turn, require large amounts of nitrogen and phosphorus, reducing the carbon-nitrogen ratio in the leaves (Agren, 2004). The leaves of functional type II species contained higher nitrogen and phosphorus contents than those of the other functional types (Figure 2), indicating that these plants can increase their growth rate by increasing their photosynthetic capacity, allowing them to eventually adapt to drought conditions. By contrast, the leaves of functional type IV plants had relatively low nitrogen and phosphorus contents but relatively high carbon contents (Figure 2), indicating that they could accumulate dry matter quite efficiently to adapt to drought conditions. Functional type V plants experienced a decrease in specific leaf area in response to drought (Figure 4E, 5E), which is speculated to increase water retention and thereby reduce transpiration and facilitate heat dissipation (Ackerly et al., 2002; Hameed et al., 2012). Finally, functional type III plants adapted to drought by increasing their leaf nitrogen content (Figure 5C), which is commonly associated with a high resource-acquisition capacity (Weih et al., 2011; Jung et al., 2014). This functional type mainly included species such as *Leymus chinensis* and *Stipa grandis*, which tend to predominate in areas with relatively abundant water (Inner Mongolia-Ningxia Joint Inspection Group of Chinese Sciences of Academy, 1985). However, the grassland in Inner Mongolia generally lacks nitrogen (Yuan et al., 2006), so the ability of these plants to obtain more nitrogen will be crucial for their adaptation to arid environments.

Numerous studies have documented that plants adapt to the environment by balancing resource utilization and allocation when resources are scarce (Gong et al., 2011; Ocheltree et al., 2016). In the present study, we found that functional type I plants exhibited a significant decrease in specific leaf area and leaf nitrogen content with an increase in aridity. A decrease in specific leaf area indicates an increased water use efficiency of plants (Ackerly et al., 2002), while a decrease in leaf nitrogen content indicates a lower nitrogen use efficiency (Yuan et al., 2006). Thus, this finding suggests that functional type I plants adapted to drought by trade-offs water use efficiency against nitrogen use efficiency. This result is consistent with the findings of Gong et al. (2011), who reported trade-offs between water use efficiency and nitrogen use efficiency in a semi-arid grassland. In areas with a high water supply, plants can improve their nitrogen use efficiency at the expense of water use efficiency. However, when water is restricted, it is a better strategy to improve water use efficiency to allow plants to increase dry matter accumulation, prolong their leaf life and complete their life cycle. Leaf thickness also tends to increase with a decrease in specific leaf area (Niinemets, 2001), further limiting the light intensity that reaches the chloroplasts inside the leaves, increasing the conduction resistance of CO_2_ in the mesophyll tissues and reducing the proportion of nitrogen that is distributed to the photosynthetic organs (Poorter and Evans, 1998), thereby decreasing the nitrogen content in the leaves.

In summary, our study demonstrated that plants in the Inner Mongolia grassland adapt to drought in four different ways including adjusting the leaf carbon-nitrogen ratio, the specific leaf area, the leaf nitrogen content, as well as the specific leaf area and leaf nitrogen content simultaneously. This finding is consistent with the LES which runs from species with conservative resource-use strategy to predominant resource-acquisition strategy (Wright et al., 2004). Functional type II, III, and IV plants adjusted their leaf nitrogen contents and carbon-nitrogen ratios and thus correspond to conservative resource-use strategy plants, while functional type V plants adjusted their specific leaf area, corresponding with predominantly resource-acquisition strategy plants, and functional type I plants adjusted their specific leaf area and nitrogen content simultaneously indicate that the trade-offs in the LES. Thus, it is clear that the LES exists in the Inner Mongolia grassland and can be applied to reveal the adaptation of plants to drought.

## Author Contributions

QZ and YD conceived the ideas. YY and YL complied the experiment and ran further data analysis. YY, YL, QL, QZ, and YD led the writing.

## Conflict of Interest Statement

The authors declare that the research was conducted in the absence of any commercial or financial relationships that could be construed as a potential conflict of interest.
